# Evaluating DNA methylation age on the Illumina MethylationEPIC Bead Chip

**DOI:** 10.1371/journal.pone.0207834

**Published:** 2019-04-19

**Authors:** Radhika Dhingra, Lydia Coulter Kwee, David Diaz-Sanchez, Robert B. Devlin, Wayne Cascio, Elizabeth R. Hauser, Simon Gregory, Svati Shah, William E. Kraus, Kenneth Olden, Cavin K. Ward-Caviness

**Affiliations:** 1 National Health and Environmental Effects Laboratory, US Environmental Protection Agency, Chapel Hill, NC, United States of America; 2 Department of Environmental Sciences and Engineering, Gillings School of Public Health, University of North Carolina, Chapel Hill, NC, United States of America; 3 Institute for Environmental Health Solutions, University of North Carolina, Chapel Hill, NC United States of America; 4 Duke Molecular Physiology Institute, Duke University Medical Center, Durham, NC, United States of America; 5 Department of Biostatistics and Bioinformatics, Duke University Medical Center, Durham, NC, United States of America; 6 Cooperative Studies Program Epidemiology Center, Durham Veterans Affairs Medical Center, Durham, NC, United States of America; 7 Division of Cardiology, Department of Medicine, School of Medicine, Duke University, Durham, NC, United States of America; 8 National Center for Environmental Assessment, US Environmental Protection Agency, Chapel Hill, NC, United States of America; Centre de Recherche en Cancerologie de Lyon, FRANCE

## Abstract

DNA methylation age (DNAm age) has become a widely utilized epigenetic biomarker for the aging process. The Horvath method for determining DNAm age is perhaps the most widely utilized and validated DNA methylation age assessment measure. Horvath DNAm age is calculated based on methylation measurements at 353 loci, present on Illumina’s 450k and 27k DNA methylation microarrays. With increasing use of the more recently developed Illumina MethylationEPIC (850k) microarray, it is worth revisiting this aging measure to evaluate estimation differences due to array design. Of the requisite 353 loci, 17 are missing from the 850k microarray. Similarly, an alternate, 71 loci DNA methylation age assessment measure created by Hannum *et al*. is missing 6 requisite loci. Using 17 datasets with 27k, 450k, and/or 850k methylation data, we compared each sample’s epigenetic age estimated from all 353 loci required by the Horvath DNAm age calculator, and using only the 336 loci available on the 850k array. In 450k/27k data, removing loci not on the 850k array resulted in underestimation of Horvath’s DNAm age. Underestimation of Horvath DNAm age increased from ages 0 to ~20, remaining stable thereafter (mean deviation = -3.46 y, SD = 1.13 for individuals ≥20 years). Underestimation of Horvath’s DNAm age by the reduced 450k/27k data was similar to the underestimation observed in the 850k data indicating it is driven by missing probes. In analogous examination of Hannum’s DNAm age, the magnitude and direction of epigenetic age misestimation varied with chronological age. In conclusion, inter-array deviations in DNAm age estimations may be largely driven by missing probes between arrays, despite default probe imputation procedures. Though correlations and associations based on Horvath’s DNAm age may be unaffected, researchers should exercise caution when interpreting results based on absolute differences in DNAm age or when mixing samples assayed on different arrays.

## Introduction

DNA methylation has recently shown promise as an accurate biomarker of aging. Recent “epigenetic clocks” developed by Horvath [1] and Hannum [2] have been shown to be an accurate estimator of age across multiple tissues and populations, and differences between DNA methylation age and chronological age are associated with pathophysiological biomarkers and incident disease [3].

The method developed by developed by Horvath [1] is perhaps the most widely used and validated epigenetic age estimation method; it relies on measurement of percent methylation at 353 loci (CpGs) on either the Illumina 450k (450k) or Illumina 27k (27k) microarray chips. The Hannum epigenetic estimation method was developed for the 450k microarray and relies on measurement of percent methylation at 71 loci. Recently, Illumina released the Infinium MethylationEPIC Bead Chip (850k), which uses the same technology as the Illumina 450K microarray to assay 866,836 CpGs [4]. Though the 850k microarray assays more loci, 8.9% of CpGs included on 450K microarray were omitted from the 850k microarray. In particular, 17 of the 353 CpGs (4.8%), and 6 of the 71 CpGs (8.5%) necessary to calculate epigenetic age via the Horvath and Hannum methods, respectively, are missing. While missing CpGs are imputed in Horvath’s online calculator [5] and can be imputed for in the Hannum method [2] to allow for estimation of epigenetic age, systematically missing probes may introduce bias in DNAm age estimates even after imputation. Biased estimates can consequently alter the detection or interpretation of associations with health outcomes and inhibit cross-platform comparisons and analyses.

To evaluate the impact of microarray design changes on the estimation of DNA methylation age, we compared the Horvath DNA methylation age (DNAm age) calculated using all 353 CpGs (full DNAm age) to estimates obtained from using either the 27k or 450k platform while restricting to the 336 CpGs available on the 850k platform. An analogous exploration was conducted for the Hannum DNAm age. We used 15 publicly available non-cancer blood tissue datasets (available in the Gene Expression Omnibus(GEO), https://www.ncbi.nlm.nih.gov/geo/), as well as blood samples from a cardiac catheterization cohort (CATHeterization GENetics; CATHGEN) where DNA methylation was assessed on both the 450k and 850k arrays.

## Methods

### Missing loci and datasets

To determine which loci in the epigenetic clocks are missing from the 850k platform we compared the 850k manifest of probe loci and the list of loci required for both Horvath’s and Hannum’s estimation of epigenetic age (available in Additional File 3 of [1] and in Table S3 of [2]).

From the 81 datasets used to develop the Horvath epigenetic clock, we selected those 15 datasets (detailed in S1 Table) whose non-cancerous samples were drawn from blood (excluding cord blood), were publicly available on the Gene Expression Omnibus (GEO; https://www.ncbi.nlm.nih.gov/geo/) and whose methylation beta values were readily available on GEO. Though chronological age was not available in GSE42865 and GSE35069, and sex was not available in GSE30870 and GSE 42865, these datasets were also included in analyses that did not require age or sex.

**Table 1:** *Missing probes annotated with SNP presence* [15], *and UCSC RefGene Name*, *chromosome and probe type*.

	CpG	SNP?	UCSC RefGene Name	Chromosome	Probe Type
**HORVATH**					
	**cg19945840**	no	SDF4; B3GALT6	1	II
	**cg02972551**	no	KDM3A	2	II
	**cg02654291**	yes	C9orf64	9	II
	**cg13682722**	yes	C14orf102	14	II
	**cg09869858**	yes	P11	12	II
	**cg06117855**	yes	CLEC3B;CLEC3B	3	II
	**cg05590257**	yes	PLD6	17	I
	**cg27016307**	yes	HRC	19	II
	**cg24471894**	yes	KIAA0020	9	II
	**cg04431054**	no	PRRC1	5	II
	**cg16494477**	no	FGF18	5	II
	**cg19046959**	no	COL8A2	1	II
	**cg17408647**	yes	C7orf44	7	II
	**cg27319898**	no	ZNF804B	7	II
	**cg19569684**	no	MGC29506	5	II
	**cg19273182**	no	PAPOLG	2	II
	**cg09785172**	no	WFS1	4	II
**HANNUM**					
	**cg24079702**	no	FHL2	2	I
	**cg07927379**	no	RNF32	7	I
	**cg21139312**	no	MSI2	17	I
	**cg14361627**	yes	KLF14	7	II
	**cg18473521**	no	HOXC4	12	II
	**cg09651136**	no	PKM2	15	II

Samples (N = 3,672) in the 15 eligible GEO datasets (summarized in S1 Table) were drawn from people ages 0 to 101, and included whole blood, peripheral blood monocytes (PBMC) and single leukocyte cell types. GSE 19711 was divided into two datasets (controls and ovarian cancer cases) for consistency with the Horvath epigenetic clock manuscript [1]. Though a few of these datasets include samples from cancer patients, the tissue obtained was non-cancerous, and their methylation age had previously shown no association to cancer [1]. Further information about these datasets may be found on GEO, and in Additional file 2 of Horvath’s manuscript which describes these datasets and their rationale for inclusion in the development of his epigenetic clock [1]. As the Hannum epigenetic clock was developed for the 450k platform and only 10% of the required loci are available on the 27k platform, we restricted all exploration of the Hannum epigenetic clock to datasets process on the 450k and 850k platforms.

In addition to the GEO datasets, two datasets from the Catheterization Genetics cohort (CATHGEN) were employed to compare the 450k and 850k platforms. CATHGEN participants were recruited from subjects undergoing an outpatient cardiac catheterization at Duke University from 2001–2011 [6]. Ethics approval was administered by the Duke Institutional Review Board for CATHGEN.

The samples were processed by reading in the idat files using minfi v1.21.1[7], examining samples for exclusion based on Illumina’s default quality control (QC) procedures, background correction via minfi’s ssNoob [7], and extracting the un-normalized beta values. The CATHGEN samples processed on the 450k and 850k microarrays were not obtained from the same individuals, and no samples were excluded based on QC for the 450k microarray, while two samples from the 850k microarray were excluded. This left 205 CATHGEN samples available from the 450k microarray (ages 23–91 y) and 568 samples from the 850k microarray (ages 33–87 y).

### DNA methylation age processing

Methylation beta values were extracted from the downloaded GEO datasets, and were not further normalized before uploading to Horavth’s (online) DNA Methylation Age Calculator as recommended (https://dnamage.genetics.ucla.edu/). The online DNA methylation age calculator automatically imputes any missing probes (https://dnamage.genetics.ucla.edu/home). Where GEO datasets were previously normalized, we deselected the normalize data option during processing in the DNA methylation calculator; otherwise, the normalize data option was selected for unnormalized data. Hannum DNA methylation age was estimated according to previously described procedures, [2] which include a k nearest neighbors imputation for missing loci. All samples were included from the publicly available data. Sex, age, sample id and blood type were extracted from the downloaded GEO datasets.

### The epigenetic clock across the age ranges in 450k/27k data

To ascertain the effect of the 17 missing loci on the estimation of epigenetic age via Horvath’s 353-probe DNA methylation clock, we calculated DNA methylation age in 27k and 450k datasets (GEO & CATHGEN 450K datasets) with and without the 17 probes unavailable on the 850k microarray. For each GEO dataset, as well as the CATHGEN 450k datasets, the Horvath DNAm age calculated using the reduced 450k data, defined as the 450k data with the probes not on the 850k array removed, was compared to Horvath DNAm age calculated using the full 450k data, graphically and using summary statistics. The comparisons were repeated in subjects chronologically aged 20 y or less, and in ages ≥ 20 y, a cutoff selected based on the observed inflection point in the plot of age vs the difference in the Horvath DNA methylation age estimated using the full and reduced 450k data, as well as the age cutoff specified in the Horvath algorithm’s transformation of chronological age (see Supporting information file 2 [1]). Analogous explorations were conducted for the Hannum DNA methylation clock, such that the Hannum DNA methylation age was estimated using the full complement of 71 loci and using the reduced complement of 65 loci that are available on the 850k clock. Because the Hannum algorithm was developed using the 450k platform and only a small fraction of the 71 loci are available on the 27k platform, analyses of the Hannum algorithm were restricted to the ten 450k and 850k datasets. Though, the Hannum DNAm age algorithm was developed in DNA methylation samples primarily from adults, we applied our analyses to all available 450k datasets, in order to facilitate comparisons between the two algorithms. Using all samples within each age group (< 20 y vs ≥ 20 y), we separately regressed full 450k (Horvath) DNAm age and the reduced 450k (Horvath) DNAm age on chronological age, and compared resulting the intercepts and chronological age slopes estimates. This analysis excluded the GSE42865 and GSE35069 datasets as chronological age was not publicly available.

Within each age group, we also assessed the possibility that the relationship between Horvath’s DNAm age, and thus age acceleration, and a variable of interest could differ based on the array type and the removal of the 17 loci from the 353 loci used to estimate Horvath DNAm age that are not available on the 850k array. As sex was the only widely available variable in the public data, we separately regressed age acceleration estimated based on the full and reduced 450k data on sex (ref. = Male), using all available samples within each age group. We repeated these analyses in each individual dataset, without regard to the chronological age of samples. We then compared the coefficient obtained from regressing Horvath age acceleration (dependent variable) on sex (independent variable) when using the full 450k data versus the reduced 450k data. Additionally, we compared residual plots of full and reduced 450k data Horvath DNAm age regressed on chronological age for all GEO datasets where age was available in the CATHGEN 450k dataset.

### Comparison of DNA methylation age in 450k and 850k datasets

The CATHGEN data were used to ascertain if technological changes in the 850k platform as compared to the 450k or 27k platforms contribute to mis-estimation of epigenetic age. To that end, *full* and *reduced* datasets for the samples processed on the 450k, as well as a dataset for the samples processed on the 850k were created for CATHGEN. Linear fits of both the Horvath and Hannum epigenetic ages by chronological age for each of the 3 CATHGEN datasets were produced. The intercept and slopes of these linear fits were compared, to ascertain if the 850k platform impacts the methylation measurement such that it would impact the calculation of epigenetic age, in a manner separate from the effect of the missing probes.

The CATHGEN dataset affords the ability to quantify any deviation of 850k DNAm ages from expected values. We regressed DNAm age on categorical variables for dataset types (full 450k and 850k in one model and reduced 450k and 850k in the second model) while controlling for age. In both models, the 450k DNAm age, either full or reduced was the referent category. These comparisons were carried out using the Horvath DNAm age estimation algorithm.

### Software and statistical analyses

All work to determine the lost loci, to prepare data for Horvath’s online DNA Methylation Age Calculator (https://dnamage.genetics.ucla.edu/), to estimate the Hannum DNA methylation age, and to subsequently compare epigenetic age estimates with chronological age were performed in R (version 3.4.0) [8] using using Bioconductor [9], minfi [10,11], IlluminaHumanMethylation450kmanifest [12], IlluminaHumanMethylationEPICmanifest [13], and impute [14] R packages.

### Terminology

Three categories of DNA methylation data were used in this analysis: 1) data from the Illumina 450k array or the 27k array (“full 450k data”); 2) data from the Illumina 450k or 27k arrays with the missing probes (17 in the Horvath clock and 6 in the Hannum clock) not on the Illumina 850k array removed (“reduced 450k data); and 3) data from the Illumina 850k array (“850k data”). “Reduced 450k DNAm age” and “full 450k DNAm age” refer to the application of the epigenetic clock to reduced and full 450k data, respectively.

## Results

### Missing probes & descriptions of the datasets

The required DNA methylation age loci that are not included in the 850k manifest are listed in Table 1. The GEO and CATHGEN 450k datasets together encompass 3,973 individuals (52% female, among those reporting sex) whose ages range from 0 (i.e., newborn) to 101 years (Table 2). In addition, we had 568 independent CATHGEN samples that were processed on the 850k platform.

**Table 2:** Comparison of Horvath’s DNA methylation age (DNAm age) estimation from full 450k data, reduced 450k data, and 850k data in GEO and CATHGEN datasets. The mean, standard deviations and correlation with chronological age (Age corr.) of DNAm age are provided for each dataset.

										(Full) 450k data (353 loci)		Reduced 450k or 850k data (336 loci)		Comparison
		Plat- form	N (prop. female)			Chronological age				DNA methylation age		DNA methylation age		(450k data DNAm age)— (red. 450k data DNAm age)
GEO Series no.						Median (range)	Mean (SD)			Mean (SD)	Age corr.	Mean (SD)	Age corr.	Mean (SD)
**HORVATH EPIGENETIC AGE ANALYSES**														
**GSE19711cases** [16,17]		27K	266 (1.0)			67 (49, 91)	66.42 (9.35)			62.5 (11.47)	0.55	58.43 (11.02)	0.56	4.11 (0.81)
**GSE19711controls** [16,17]		27K	274 (1.0)			64 (52, 78)	64.89 (6.74)			62.57 (7.65)	0.66	58.56 (7.52)	0.66	4.01 (0.68)
**GSE20067** [16,18]		27K	192 (0.51)			43 (24,74)	43.9 (9.8)			43.45 (9.27)	0.81	38.55 (9.2)	0.81	4.85 (0.95)
**GSE20236** [19]		27K	93 (1.0)			63 (49,74)	62.86 (6.33)			53.79 (6.51)	0.69	49.92 (6.32)	0.68	3.87 (0.58)
**GSE20242** [19]		27K	50 (0.74)			34 (16,69)	35.86 (13.89)			45.02 (27.45)	0.55	41.49 (27.71)	0.53	2.30 (0.84)
**GSE27097** [20]		27K	398 (0.0)			9.3 (3.6, 17.8)	9.89 (3.63)			9.6 (4.41)	0.75	8.14 (3.88)	0.72	1.46 (0.69)
**GSE30870** [21]**		450K	38 (0.74)			44.5 (0, 100)	46.32 (47.01)			41.06 (42.02)	0.99	38.93 (39.95)	0.99	2.14 (2.13)
**GSE32149** [22]		450K	48 (0.52)			15 (3.5,76)	22.15 (18.43)			22.3 (15.13)	0.96	19.96 (14.34)	0.97	2.34 (0.92)
**GSE35069** [23]*		450K	60 (0.0)			NA	NA			41.74 (12.75)	-	39.15 (12.84)	-	2.59 (0.56)
**GSE36064** [20]		450K	78 (0.0)			3.1 (1.0, 16.1)	4.58 (4.11)			4.38 (3.92)	0.93	3.62 (3.27)	0.93	0.76 (0.66)
**GSE40279** [2]		450K	656 (0.52)			65 (19, 101)	64.04 (14.74)			63.08 (11.53)	0.91	60.67 (11.66)	0.92	2.41 (0.70)
**GSE41037** [24]		27K	720 (0.38)			33 (16, 88)	37.4 (15.72)			36.85 (15.38)	0.95	33.07 (15.07)	0.96	3.81 (0.79)
**GSE41169** [24]		450K	95 (0.29)			29 (18, 65)	31.57 (10.28)			31.23 (11.01)	0.94	27.67 (10.69)	0.94	3.55 (0.60)
**GSE42861** [25]		450K	689 (0.71)			54 (18, 70)	51.93 (11.8)			53.38 (11.09)	0.90	50.22 (11.01)	0.90	3.16 (0.58)
**GSE42865** [26]* **		450K	15 (0.62)			NA	NA			38.19 (9.45)	-	35.68 (9.68)	-	2.40 (1.10)
**CATHGEN 450k** ˚		450k	206 (0.37)			64 (33,87)	63.41 (11.85)			64.58 (10.50)	0.88	60.73 (10.23)	0.87	3.85 (0.72)
**CATHGEN 850k** **†˚**		850k	568 (0.41)			59 (23, 91)	60.11 (12.44)			-	-	58.16 (10.51)	0.86	-
**HANNUM EPIGENETIC AGE ANALYSES**														
**GSE30870** [21]**	450K			38 (0.74)	44.5 (0, 100)			46.32 (47.01)	43.69 (49.27)		0.99	44.82 (43.83)	0.99	-1.13 (5.48)
**GSE32149** [22]	450K			48 (0.52)	15 (3.5,76)			22.15 (18.43)	27.11 (16.16)		0.89	29.92 (14.13)	0.90	-2.82 (2.18)
**GSE35069** [23]*	450K			60 (0.0)	NA			NA	45.91 (15.79)		-	46.05 (14.16)	-	-0.15 (1.89)
**GSE36064** [20]	450K			78 (0.0)	3.1 (1.0, 16.1)			4.58 (4.11)	3.97 (8.28)		0.88	9.81 (7.3)	0.88	-5.84 (1.15)
**GSE40279** [2]	450K			656 (0.52)	65 (19, 101)			64.04 (14.74)	68.78 (13.11)		0.95	66.9 (11.87)	0.95	1.88 (1.54)
**GSE41169** [24]	450K			95 (0.29)	29 (18, 65)			31.57 (10.28)	31.91 (11.14)		0.93	33.47 (9.81)	0.93	-1.56 (1.51)
**GSE42861** [25]	450K			689 (0.71)	54 (18, 70)			51.93 (11.8)	59.03 (10.6)		0.92	58.2 (9.49)	0.92	0.83 (1.38)
**GSE42865** [26]* **	450K			15 (0.62)	NA			NA	54.69 (15.68)		-	56.24 (15.99)	-	-1.55 (2.65)
**CATHGEN 450k** ˚	450k			206 (0.37)	64 (33,87)			63.41 (11.85)	69.04 (11.3)		0.9	62.78 (10.27)	0.90	6.26 (1.45)
**CATHGEN 850k** **†˚**	850k			568 (0.41)	59 (23, 91)			60.11 (12.44)	55.29 (11.83)		0.9	55.29 (11.83)	0.90	-

* As chronological age was missing for these datasets, correlation with age and age acceleration could not be determined.

** Proportion Female was obtained from supplemental table of the original epigenetic clock manuscript (Horvath, 2013), and were not available in GEO.

† Because the 17 loci required to complete the epigenetic clock are unavailable on the 850k platform, there is not information for the full epigenetic clock

˚ CATHGEN 450k and CATHGEN 850k are not comprised of the same individuals. That is, the underlying sample population is non-overlapping.

### Comparison of DNA methylation age in 450k data and 850k data

**Fig 1:**
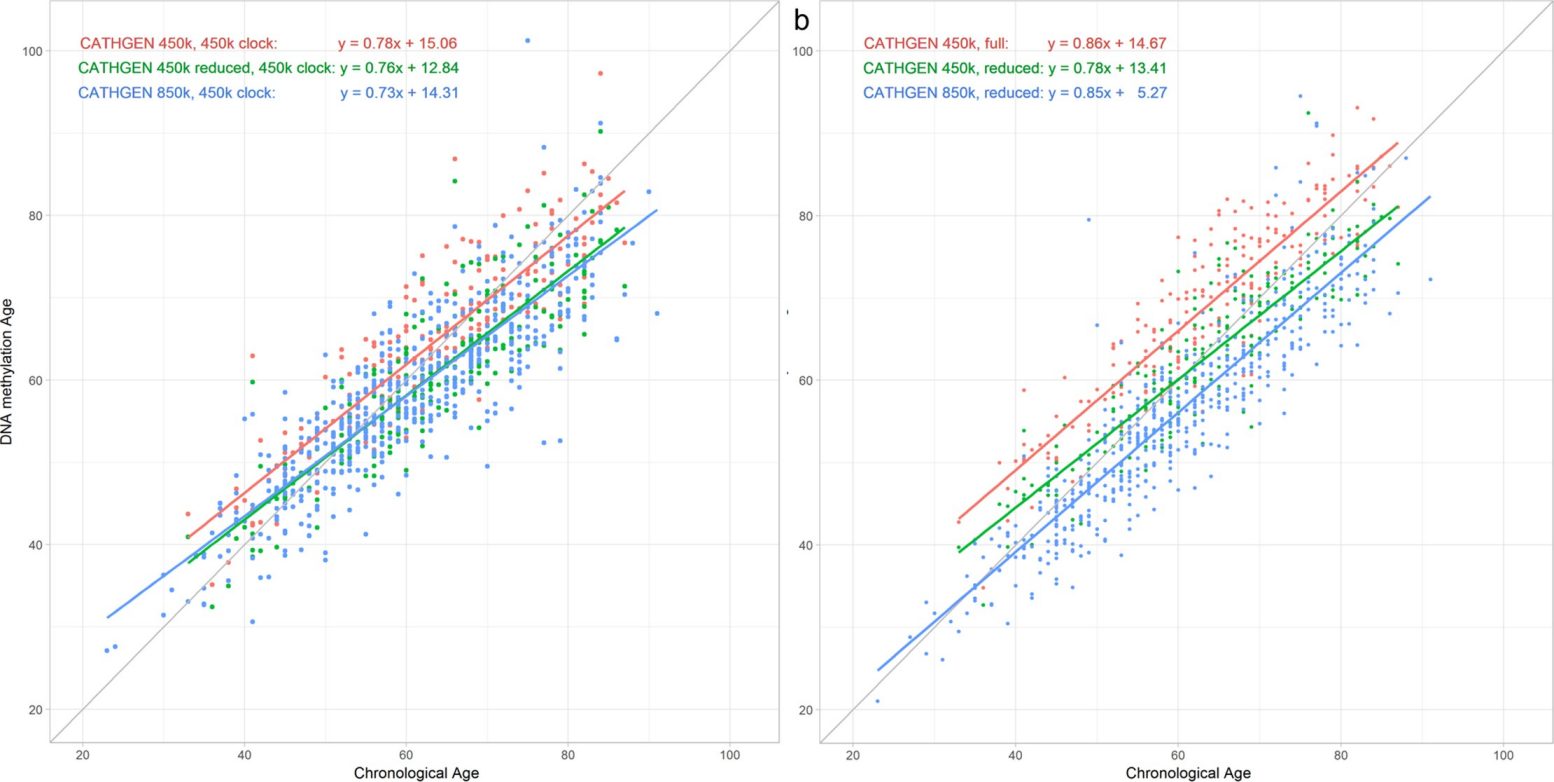
Epigenetic age by chronological age in combinations of CATHGEN dataset and epigenetic clock. The plot of DNA methylation by chronological age shows the impact of the missing probes, by applying the (a) Horvath and (b) Hannum epigenetic clocks to CATHGEN 450k ('full' and 'reduced') and 850k datasets.

The Horvath DNAm age estimated separately in the CATHGEN’s *full* 450k, *reduced* 450k and 850k datasets using the epigenetic clock all showed positive correlations with chronological age (Table 2, Fig 1). For each of these three datasets, the slope between Horvath DNAm age and chronological age is nearly identical (0.73–0.78). However, in a regression of Horvath DNAm age on dataset type (full 450k vs. 850k) correcting for age, 850k DNAm ages had a mean difference of -3.96 y (95%CI: -4.08, -3.12; p <0.0001) as compared to the full 450k, which is very close to the underestimation seen with the when comparing CATHGEN DNAm age estimates from the reduced 450k data with the full 450k data (paired t-test: 3.85 y, p<0.0001). There was no significant difference between the 850k DNAm age and reduced 450k DNAm age in CATHGEN (-0.14; 95%CI: -0.98, 0.70, p = 0.75).

The Hannum DNAm age in CATHGEN’s full 450k dataset is greater than chronological age (indicating accelerated aging, Fig 1), but in the CATHGEN 850k data Hannum DNAm age is lower than chronological age, though these are different samples from the 450k data. The slopes between Hannum DNAm age and chronological age for the full 450k and the 850k datasets are nearly identical (0.86 and 0.85, respectively), while their intercepts differ by 9.4 years. Hannum DNAm Age in the reduced 450k dataset falls between these two regression lines with an intercept that is closest to that of the full 450k data, but with a significantly different slope of 0.78 (p = 0.04).

### Probe exclusion effects on Horvath DNAm age in 16 datasets

**Fig 2:**
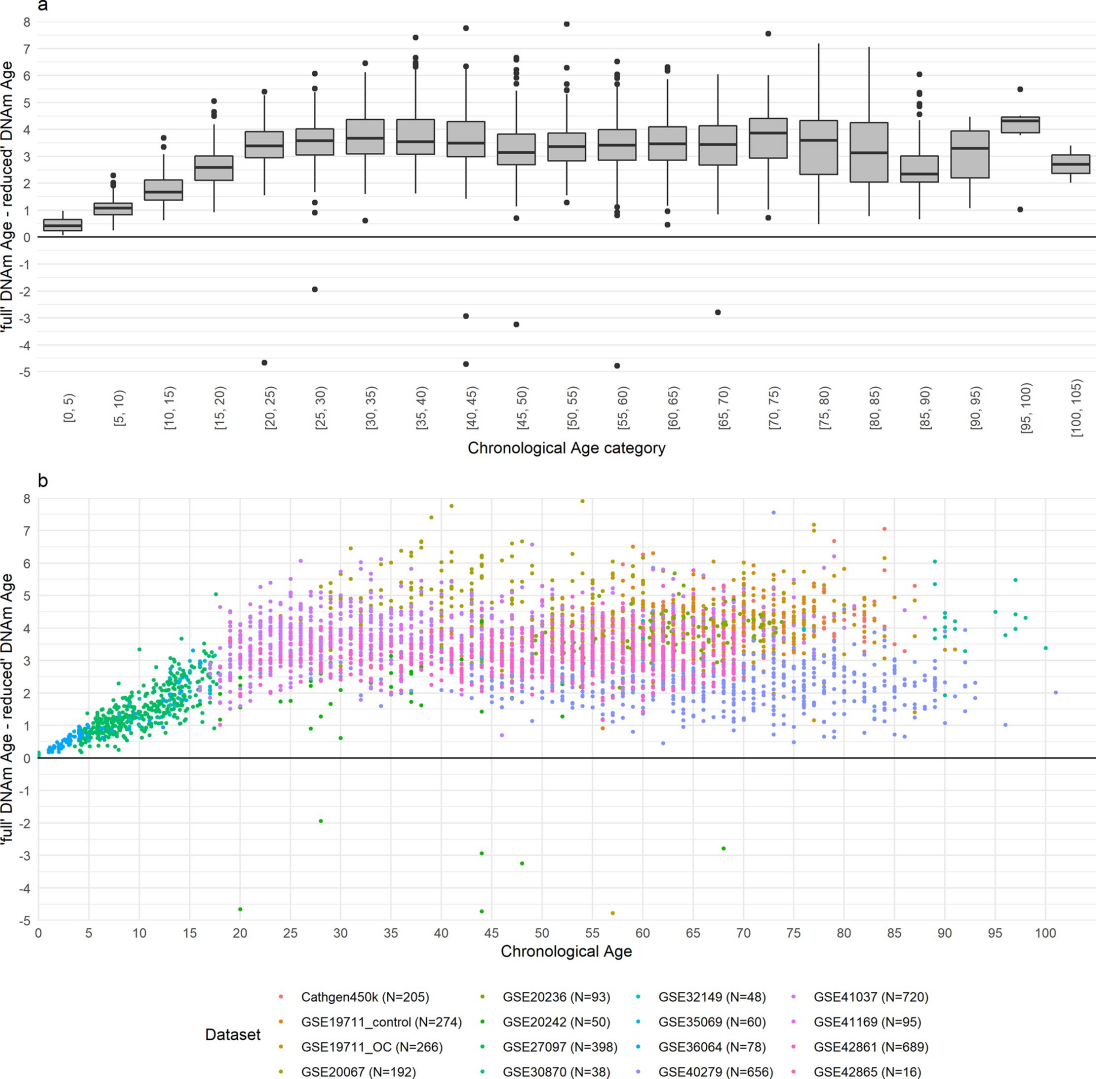
Difference of 'full' and 'reduced' Horvath epigenetic Age by chronological age. The difference of ‘full and reduced’ epigenetic ages calculated in the GEO (450k and 27k) and CATHGEN 450k data are presented as (a) boxplot by 5 year chronological age categories and (b) as a scatterplot.

Across all 16 datasets with 450k or 27k data, reduced 450k Horvath DNAm age underestimated the Horvath full 450k DNAm age (S1 Fig). In peripheral blood samples from the youngest individuals (chronological age < 20 y), the individual difference between epigenetic age as estimated using the *full* and *reduced* datasets increased with age (Fig 2, Table 3). However, in samples from older individuals, (chronological age ≥ 20 y), the difference did not increase with age but we observed greater inter-individual variability in the difference between full and reduced DNAm age in older individuals (SD = 1.13) than in the younger age group (ages 0-5y: SD = 0.27; ages 5-10y: 0.35; ages 10-15y: SD = 0.54; and ages 15-20y: SD = 0.82). Across all datasets, the correlation between full and reduced 450k data remained high ranging from 0.989 to 0.999.

**Table 3:** Regression of Horvath DNA methylation age on chronological age, by age group, in the full and reduced 450k/27k datasets (GEO and CATHGEN).

	Age < 20 years (N = 616)		Age ≥ 20 years (N = 2,972)	
	Intercept	Chronological Age	Intercept	Chronological Age
Data	Estimate (95% CI)	Estimate (95% CI)	Estimate (95% CI)	Estimate (95% CI)
**'full' 450k/27k**	-0.28 (-1.03, 0.47)	1.02 (0.96, 1.09)	7.02 (6.25, 7.93)	0.85 (0.84, 0.87)
**'reduced' 450k/27k**	-0.32 (-1.09, 0.45)	0.88 (0.81, 0.94)	3.18 (2.40, 3.95)	0.86 (0.85, 0.88)

Regressions of Horvath DNAm age on chronological age within the full and reduced datasets, within each age group, reveal further age-dependent differences (Table 3). Among those with age <20 y, the slope in the reduced datasets is shallower and significantly differ (t-test, p = 0.002) when compared with the full dataset, while the intercepts do not differ (t-test, p = 0.94). Among those ≥ 20 years old, the slopes do not differ significantly (t-test, p = 0.84), but the underestimation of DNA methylation age by the reduced data, as compared to the full data, is 3.84 y (t-test, p<0.001) at the intercept.

### Probe exclusion effects on Hannum DNAm age in 10 datasets

**Fig 3:**
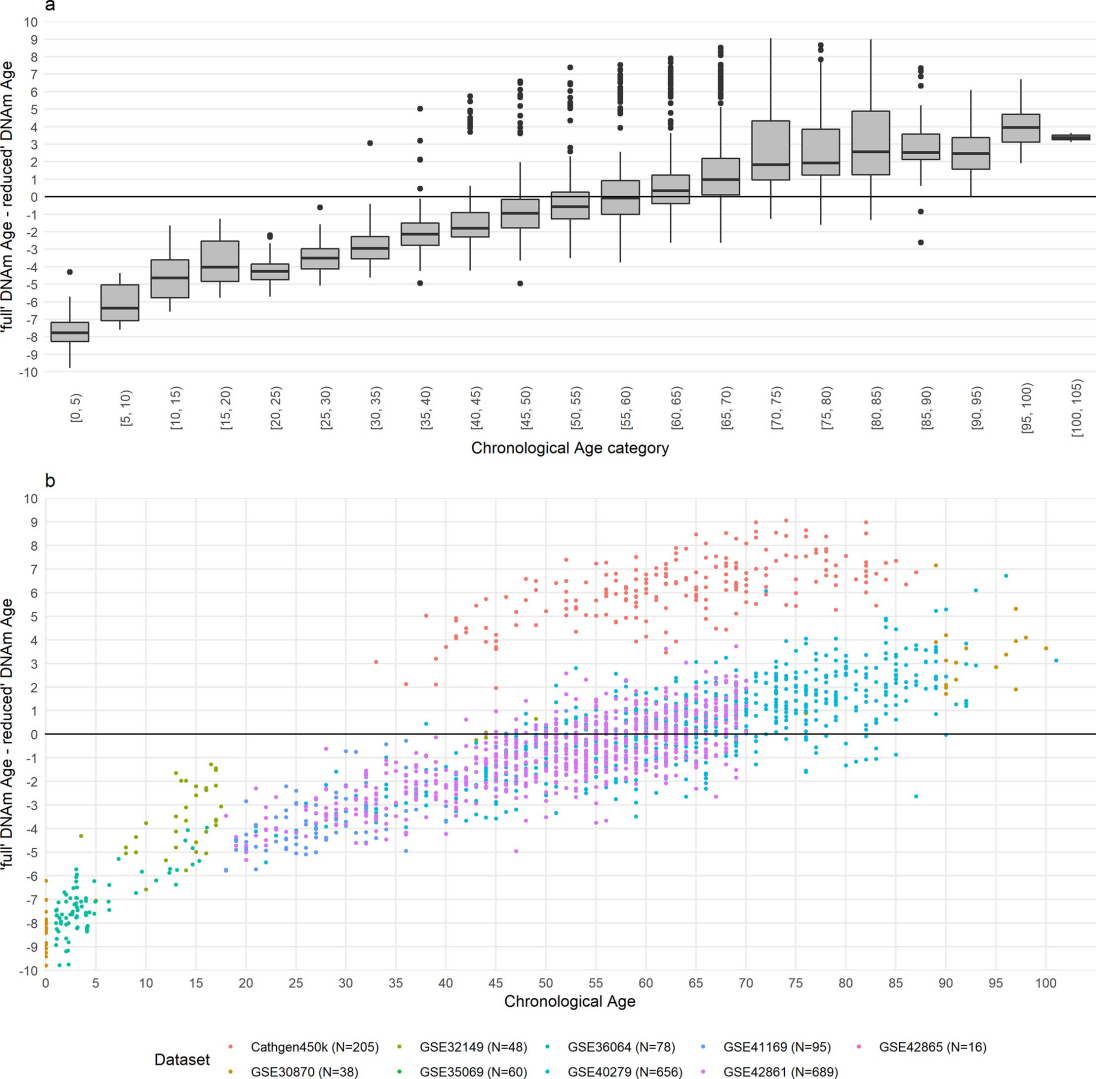
Difference of 'full' and 'reduced' Hannum epigenetic Age by chronological age. The difference of ‘full and reduced’ epigenetic ages calculated in the GEO (450k and 27k) and CATHGEN 450k data are presented as (a) boxplot by 5-year chronological age categories and (b) as a scatterplot.

The misestimation of Hannum DNAm age in reduced as compared to full 450k data varies with age (Table2, Fig 3). In peripheral blood samples from younger individuals (<40 years), the Hannum algorithm overestimated the DNAm age in the reduced vs. the full 450k data. In contrast, Hannum DNAm age are largely underestimated in reduced 450k dataset as compared to full 450k data amongst individuals over 60 years of age.

### Potential impact of underestimation in Horvath DNAm age on regression outcomes

If the misestimation of DNAm age due to missing probes is uniform across age ranges then associations between DNAm age and a variable of interest should be comparable in the reduced and full 450k datasets. Given the differences in Horvath DNAm age estimation for individuals age <20 y vs. ≥20 y (Table 3, S1 Fig), we examined associations between age acceleration and sex in both age groups (Table 4). Using DNAm age acceleration, the residuals of age regressed on Horvath DNAm age, the effect estimates obtained in the full 450k data were not significantly different from those obtained in the reduced 450k data in subjects aged 20 years or more (p = 0.87) nor in subjects <20 years (p = 0.22). This finding did not differ when we used Horvath DNAm age in place of the age acceleration measure (not shown), and did not differ depending on whether the data was derived from the 27k array or 450k array. Residual violin plots for regressions of DNAm age on sex (S2 Fig) show no large or systematic differences in the distribution of DNAm age residuals, further reinforcing the similarity of the regressions with and without the removal of the 17 probes missing from the 850k platform.

**Table 4:** Regressions of age acceleration on sex for CATHGEN450k and GEO datasets, using Horvath DNA methylation age calculated using the (full) 450k data and reduced 450k data. Regressions were conducted for each dataset individually, and then in aggregate while stratifying for chronological age (<20y and ≥20y). P-values result from a t-test to compare the slopes for regressions using the various DNAm ages.

	N (prop. female)	(Full) 450k/27k data DNAm age	Reduced 450k/27k data DNAm age	Full vs. reduced 450k/27k data
Dataset		Slope Est. (95%CI)	Slope Est. (95%CI)	p value
**Cathgen450k**	205 (0.38)	0.28 (-1.31, 1.87)	0.39 (-1.24, 2.02)	0.92
**GSE20067**	192 (0.51)	0.03 (-1.64, 1.7)	0.12 (-1.55, 1.8)	0.94
**GSE20242**	50 (0.74)	-3.31 (-17.88, 11.27)	-1.58 (-16.68, 13.51)	0.87
**GSE32149**	48 (0.52)	1.89 (-1.58, 5.37)	2.25 (-1.45, 5.96)	0.89
**GSE40279**	656 (0.52)	1.41 (0.46, 2.36)	1.39 (0.45, 2.33)	0.98
**GSE41037**	720 (0.38)	1.25 (0.53, 1.97)	1.04 (0.36, 1.73)	0.68
**GSE41169**	95 (0.29)	-0.89 (-2.57, 0.78)	-1.13 (-2.72, 0.46)	0.84
**GSE42861**	689 (0.71)	0.17 (-0.69, 1.03)	0.01 (-0.85, 0.87)	0.80
**less than 20y**	662 (0.06)	-0.6 (-1.52, 0.32)	0.19 (-0.69, 1.08)	0.22
**20y or older**	3,294 (0.60)	1.51 (1.01, 2.01)	1.57 (1.07, 2.07)	0.87

## Discussion

Estimation of DNAm age is a methylation array dependent procedure, in so much as differing arrays may not have all probes used to develop the DNAm age estimator. Use of the epigenetic clock to estimate DNAm age from data generated from the Illumina MethylationEPIC array is likely to produce substantial underestimation of Horvath DNAm age or misestimation of Hannum DNAm age, relative to the DNAm age estimated with the Illumina 450K array. A 3.3-year and 5-year increased DNAm age using the Horvath epigenetic clock has been associated with an increase of 10 body mass index units [27] and a 16% increase in mortality [28], respectively. Thus, observed underestimations, in the range of 4 years, could cause substantial mis-estimations of health risks based on the Horvath DNAm age if array differences are not taken into account. Using age-adjusted residuals (Horvath DNA methylation age acceleration) or adjusting for age when using Δage (Horvath DNAm age–chronological age) as a predictor is recommended, since the correlation between chronological age and Horvath DNA methylation age appears to be independent of array. Systematic differences due to array design would alter the intercept in such models but not regression coefficients. Thus, regression models using the Horvath DNAm age or derivative metrics will reflect highly concordant results across arrays, but this will not necessarily be reflected in comparisons of absolute epigenetic aging differences with outcomes across methylation platforms. Estimating Horvath DNAm age on a “reduced” 450k dataset (i.e., using probes only available on the 850k array) produced similar underestimation as observed when using the 850k data, indicating that the observed underestimation is primarily driven by the 17 missing probes (Table 1), as opposed to other design differences between the 850k and the 450k arrays. This might be expected given the fact that the probes used for the 850k array used the same chemistry and color channels as previous probes.

The interpretation of Hannum DNAm age results are less clear, in that the Hannum DNAm age estimated in the reduced 450k dataset produces a underestimation that varied with chronological age and differed from that observed in the 850k dataset (Fig 1). The Hannum DNAm age measure is closely correlated with blood immune cell counts which are also known to vary with age, with Horvath DNAm age is largely independent of blood immune cell counts. This linkage to immune cell counts might account for the age-dependence in the mis-estimation based on probe removal as well as the differences in mis-estimation for the Hannum versus Horvath DNAm age estimates. As the misestimation of Hannum DNAm age varies with chronological age, cross-array comparisons of results based on Hannum DNAm age will require more nuanced consideration, especially if the outcome or exposure of interest is age-related.

This study employed many of the same publicly available GEO datasets used to develop Horvath’s 450k clock, allowing direct comparisons in datasets which have been previously shown to estimate the Horvath DNAm age well [1]. We focused on blood, since that is the tissue for which the Horvath epigenetic age estimator provides the most accurate and consistent associations, and in which the Horvath DNAm age estimator has been most widely applied. Because CATHGEN 450k and 850k data were estimated on independent (i.e., non-overlapping) groups of individuals, direct comparison of the underestimation of DNAm age within individuals was not possible. However, the size of the CATHGEN datasets still offer the ability to compare these measures in the same source population, and both datasets were similar in age and sex makeup (Table 1).

The Illumina MethylationEPIC array represents a substantial step forward in the genome-wide assessment of DNA methylation. As DNA methylation array technology has progressed, researchers may wish to combine epigenetic age derived from 450k/27k and 850k data; however, the deviation in DNAm age estimates among the array platform generations may introduce error into subsequent analyses. Thus, care should be taken when using epigenetic biomarkers, such as Horvath’s clock, that were developed using 450k and 27k data, as they may not be fully optimized for the Illumina MethylationEPIC array.

## Supporting information

S1 Table Summary of GEO datasets. (DOCX)

S1 Fig Plot of reduced 450k Horvath DNA methylation age by 450k data Horvath DNA methylation age. Data are shown for CATHGEN 450k datasets and the publicly available datasets for (a) all observations, (b) those < 20 years of age, and (c) those ≥ 20 years of age. As can be seen across the plots, although the slope between the full and reduced Horvath DNA methylation age differs between the two age groups the overall correlation remains high.
(PDF)

S2 Fig Violin plots of residuals by sex, from regression of Horvath DNA methylation age acceleration on sex. Plots presented for 450k data, reduced 450k data, in the CATHGEN 450k and publicly available GEO datasets. The distribution of residuals from the regression of age acceleration on sex is the same even after removing the 17 probes, indicating that regressions using age acceleration from the reduced 450k data (which underestimates DNA methylation age) remain valid as the underestimation is captured as an intercept shift in the models.
(PDF)
