# Supplementary material for: Evaluating DNA methylation age on the Illumina MethylationEPIC Bead Chip
Source: PLoS One. 2019 Apr 19;14(4):e0207834. doi: 10.1371/journal.pone.0207834 (PMC6474589; doi:10.1371/journal.pone.0207834)
Supplement: S1 Table — (DOCX) [file pone.0207834.s001.docx]

Table S1. Summary of GEO datasets.

| **GEO Series no.** | **Plat-form** | **N (prop. female)** | **Median age (range)** | **Blood Type** | **Target population** | **Relationship of outcome (if any) to Horvath DNAm age acceleration  or to Horvath DNA Methylation** |
| --- | --- | --- | --- | --- | --- | --- |
| **GSE19711.cases** [1,2] | 27K | 266 (1.0) | 67 (49, 91) | Whole blood | Postmenopausal women, cases in study of ovarian cancer | None found |
| **GSE19711.controls** [1,2] | 27K | 274 (1.0) | 64 (52, 78) | Whole blood | Postmenopausal women, healthy controls in study of ovarian cancer |  |
| **GSE20067** [1,3] | 27K | 192 (0.51) | 43 (24,74) | Whole blood | Type 1 diabetics | NA° |
| **GSE20236** [4] | 27K | 93 (1.0) | 63 (49,74) | Whole Blood | Healthy females | NA |
| **GSE20242** [4] | 27K | 50 (0.74) | 34 (16,69) | CD4+ T-cells & CD14 monocytes | Healthy subjects | NA |
| **GSE27097** [5] | 27K | 398 (0.0) | 9.3 (3.6, 17.8) | leukocytes | Healthy children | NA |
| **GSE30870** [6] | 450K | 38 (0.74) | 44.5 (0, 100) | PBMC* | Healthy newborns and nonagenarians | NA |
| **GSE32149** [7] | 450K | 48 (0.52) | 15 (3.5,76) | Peripheral blood leukocyte | Cases and healthy controls  from study of Crohn’s disease | None found [8] |
| **GSE35069** [9] | 450K | 60 (0.0) | NA | Blood cell  types ** | Individual cell types from  6 healthy male donors | NA |
| **GSE36064** [5] | 450K | 78 (0.0) | 3.1 (1.0, 16.1) | Leukocytes | Healthy male children | NA |
| **GSE40279** [10] | 450K | 656 (0.52) | 65 (19, 101) | Whole blood | Healthy individuals; previously used in Hannum’s study of methylation age | NA |
| **GSE41037** [11] | 27K | 720 (0.38) | 33 (16, 88) | Whole blood | Cases and healthy controls  in a study of schizophrenia | None found [8,12] |
| **GSE41169** [11] | 450K | 95 (0.29) | 29 (18, 65) | Whole blood | Cases and healthy controls  in a study of schizophrenia | None found [8,12] |
| **GSE42861** [13] | 450K | 689 (0.71) | 54 (18, 70) | Whole blood | Cases and controls from a  study of rheumatoid arthritis | RA associated with decrease in age acceleration of 1.2 y (p = 0.005) |
| **GSE42865** [14] | 450K | 15 (0.62) | NA | Immortalized & naïve B cells, & PBMC | Cases and controls in study of Progeria and Werner’s syndrome | None found [8] |
| * PBMC = peripheral blood mononuclear cells | | | | | | |
| ** Blood cell types included PBMC, CD4+T cells, CD8+ T cells, CD56+ NK cells, CD19+ B cells, CD14+ monocytes, neutrophils, and eosinophils  ° Not assessed, as all samples were cases. | | | | | | |

## Bibliography

1. Teschendorff AE, Menon U, Gentry-Maharaj A, Ramus SJ, Weisenberger DJ, Shen H, et al. Age-dependent DNA methylation of genes that are suppressed in stem cells is a hallmark of cancer. Genome Res. 2010;20:440–6.

2. Song H, Ramus SJ, Tyrer J, Bolton KL, Gentry-Maharaj A, Wozniak E, et al. A genome-wide association study identifies a new ovarian cancer susceptibility locus on 9p22.2. Nat. Genet. 2009;41:996–1000.

3. Bell CG, Teschendorff AE, Rakyan VK, Maxwell AP, Beck S, Savage DA. Genome-wide DNA methylation analysis for diabetic nephropathy in type 1 diabetes mellitus. BMC Med. Genomics [Internet]. 2010;3:33. Available from: http://bmcmedgenomics.biomedcentral.com/articles/10.1186/1755-8794-3-33

4. Rakyan VK, Down TA, Maslau S, Andrew T, Yang TP, Beyan H, et al. Human aging-associated DNA hypermethylation occurs preferentially at bivalent chromatin domains. Genome Res. 2010;20:434–9.

5. Alisch RS, Barwick BG, Chopra P, Myrick LK, Satten GA, Conneely KN, et al. Age-associated DNA methylation in pediatric populations Age-associated DNA methylation in pediatric populations. Genome Res. 2012;22:623–32.

6. Heyn H, Li N, Ferreira H, Moran S, Pisano D, Gomez A, et al. Distinct DNA methylomes of newborns and centenarians. PNAS. 2012;109:10522–7.

7. Harris RA, Nagy-Szakal D, Pedersen N, Opekun A, Bronsky J, Munkholm P, et al. Genome-wide peripheral blood leukocyte DNA methylation microarrays identified a single association with inflammatory bowel diseases. Inflamm Bowel Dis. 2012;18:2334–41.

8. Horvath S. DNA methylation age of human tissues and cell types. Genome Biol. [Internet]. 2013;14:R115. Available from: http://genomebiology.com/2013/14/10/R115

9. Reinius LE, Acevedo N, Joerink M, Pershagen G, Dahlén SE, Greco D, et al. Differential DNA methylation in purified human blood cells: Implications for cell lineage and studies on disease susceptibility. PLoS One. 2012;7:e41361.

10. Hannum G, Guinney J, Zhao L, Zhang L, Hughes G, Sadda S, et al. Genome-wide Methylation Profiles Reveal Quantitative Views of Human Aging Rates. Mol. Cell. Elsevier Inc.; 2013;49:359–67.

11. Horvath S, Zhang Y, Langfelder P, Kahn RS, Boks MPM, Eijk K Van, et al. Aging effects on DNA methylation modules in human brain and blood tissue. Genome Biol. [Internet]. BioMed Central Ltd; 2012;13:R97. Available from: http://genomebiology.com/2012/13/10/R97

12. Horvath S, Zhang Y, Langfelder P, Kahn RS, Boks MP, Van EK, et al. Aging effects on DNA methylation modules in human brain and blood tissue. Genome Biol. 2012;13:1465–6914.

13. Liu Y, Aryee MJ, Padyukov L, Fallin MD, Hesselberg E, Runarsson A, et al. Epigenome-wide association data implicate DNA methylation as an intermediary of genetic risk in rheumatoid arthritis. Nat. Biotechnol. 2013;31:142–7.

14. Heyn H, Moran S, Esteller M. Aberrant DNA methylation profiles in the premature aging disorders Hutchinson-Gilford Progeria and Werner Syndrome. Epigenetics. 2013;8:28–33.
