# Supplementary material for: Evaluating DNA methylation age on the Illumina MethylationEPIC Bead Chip
Source: PLoS One. 2019 Apr 19;14(4):e0207834. doi: 10.1371/journal.pone.0207834 (PMC6474589; doi:10.1371/journal.pone.0207834)
Supplement: S1 Fig — Data are shown for CATHGEN 450k datasets and the publicly available datasets for (a) all observations, (b) those < 20 years of age, and (c) those ≥ 20 years of age. As can be seen across the plots, although the slope between the full and reduced Horvath DNA methylation age differs between the two age groups the overall correlation remains high. (PDF) [file pone.0207834.s002.pdf]

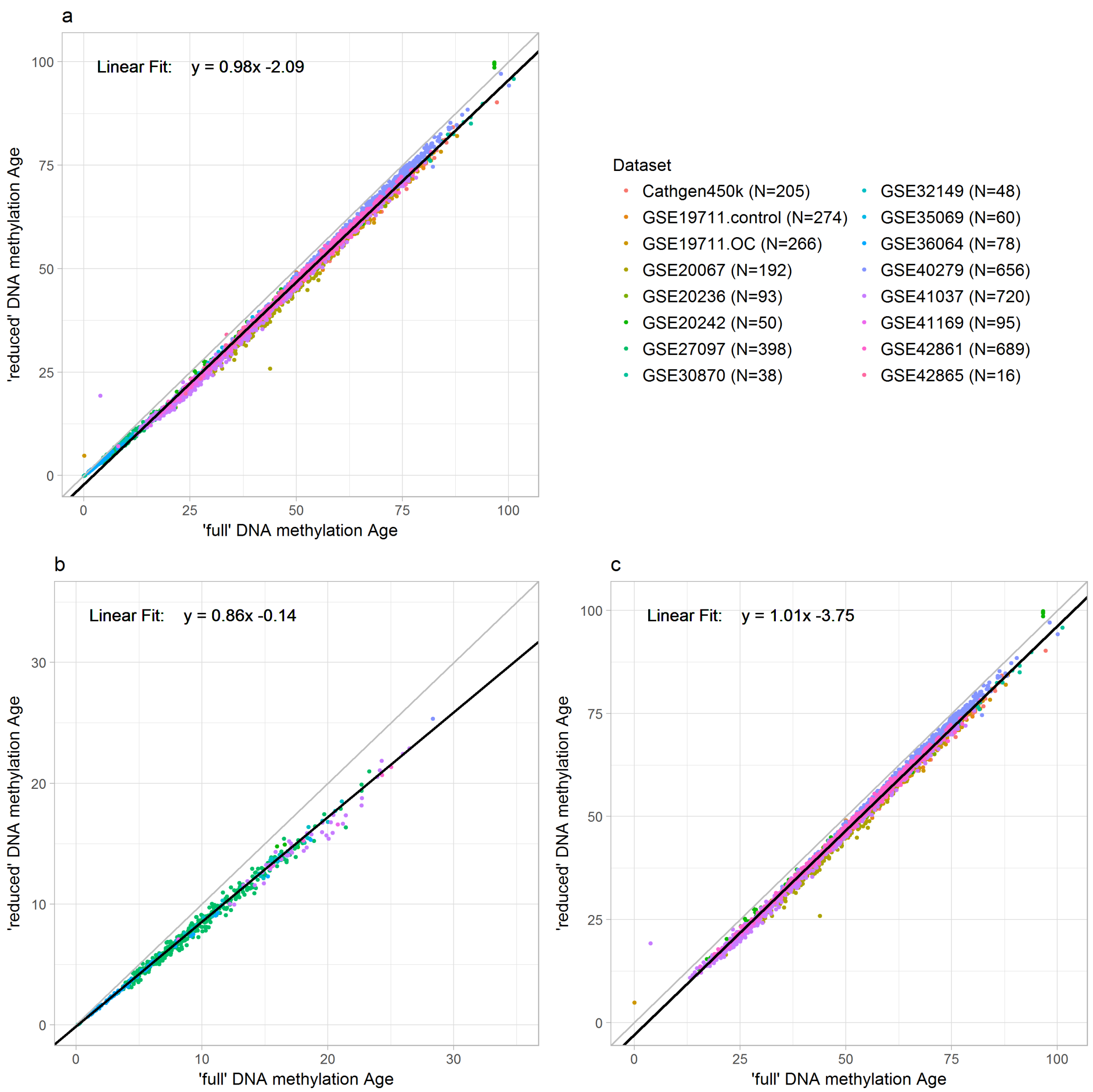

Figure S1. Plot of reduced 450k Horvath DNA methylation age by 450k data Horvath DNA methylation age*.* *Data are shown for CATHGEN 450k datasets and the publicly available datasets for (a) all observations, (b) those < 20 years of age, and (c) those ≥ 20 years of age. As can be seen across the plots, although the slope between the full and reduced Horvath DNA methylation age differs between the two age groups the overall correlation remains high.*
