# Supplementary material for: Evaluating DNA methylation age on the Illumina MethylationEPIC Bead Chip
Source: PLoS One. 2019 Apr 19;14(4):e0207834. doi: 10.1371/journal.pone.0207834 (PMC6474589; doi:10.1371/journal.pone.0207834)
Supplement: S2 Fig — Plots presented for 450k data, reduced 450k data, in the CATHGEN 450k and publicly available GEO datasets. The distribution of residuals from the regression of age acceleration on sex is the same even after removing the 17 probes, indicating that regressions using age acceleration from the reduced 450k data (which underestimates DNA methylation age) remain valid as the underestimation is captured as an intercept shift in the models. (PDF) [file pone.0207834.s003.pdf]

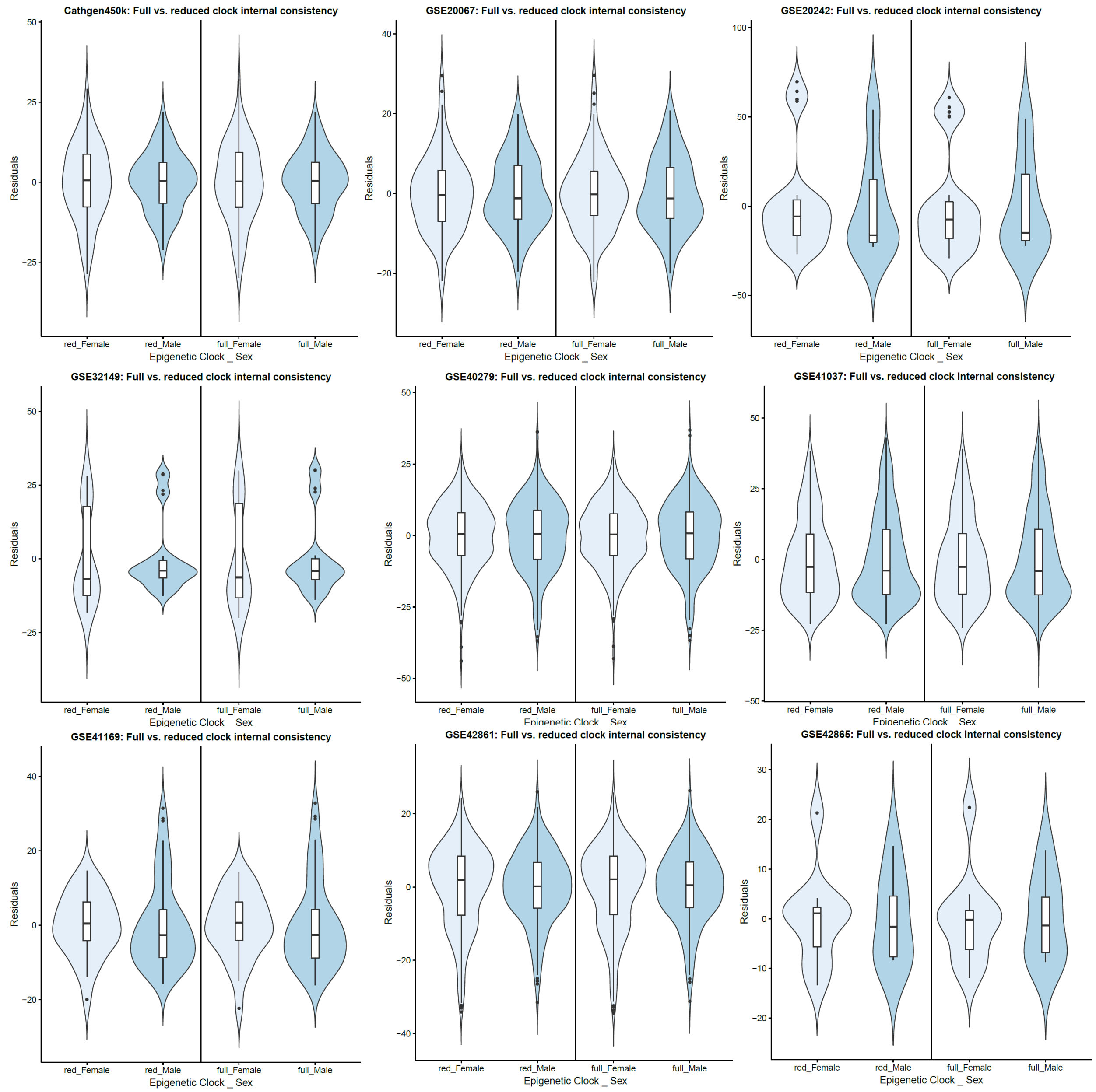

S1 Fig. Violin plots of residuals by sex, from regression of Horvath DNA methylation age acceleration on sex. *Plots presented for 450k data, reduced 450k data, in the CATHGEN 450k and publicly available GEO datasets. The distribution of residuals from the regression of age acceleration on sex is the same even after removing the 17 probes, indicating that regressions using age acceleration from the reduced 450k data (which underestimates DNA methylation age) remain valid as the underestimation is captured as an intercept shift in the models.*
